# Histometabolic Tumor Imaging of Hypoxia in Oral Cancer: Clinicopathological Correlation for Prediction of an Aggressive Phenotype

**DOI:** 10.3389/fonc.2020.01670

**Published:** 2020-08-27

**Authors:** Grégoire B. Morand, Martina A. Broglie, Paul Schumann, Martin W. Huellner, Niels J. Rupp

**Affiliations:** ^1^Department of Otorhinolaryngology, Head and Neck Surgery, University Hospital Zurich, Zurich, Switzerland; ^2^Faculty of Medicine, University of Zurich, Zurich, Switzerland; ^3^Department of Cranio-Maxillo-Facial and Oral Surgery, University Hospital Zurich, Zurich, Switzerland; ^4^Department of Nuclear Medicine, University Hospital Zurich, Zurich, Switzerland; ^5^Department of Pathology and Molecular Pathology, University Hospital Zurich, Zurich, Switzerland

**Keywords:** carcinoma, squamous cell, fluorodeoxyglucose, tumor hypoxia, mouth neoplasms, positron emission tomography, glucose transporter type 1, lymph nodes

## Abstract

**Introduction:**

Fluorodeoxyglucose-positron emission tomography (FDG-PET) is a widely used imaging tool for oral squamous cell carcinoma (OSCC). Preliminary studies indicate that quantification of tumor metabolic uptake may correlate with tumor hypoxia and aggressive phenotypes.

**Methods:**

Retrospective review of a consecutive cohort of OSCC (*n* = 98) with available pretherapeutic FDG-PET/CT, treated at the University Hospital Zurich. Clinico-pathologico-radiological correlation between maximum standard uptake value (SUV_max_) of the primary tumor, immunohistochemical staining for hypoxia-related proteins glucose transporter 1 (GLUT1) and hypoxia-inducible factor 1-alpha (HIF1a), depth of invasion (DOI), lymph node metastasis, and outcome was examined.

**Results:**

Positive staining for GLUT1 and HIF1a on immunohistopathological analysis correlated with increased SUV_max_ on pretherapeutic imaging and with increased DOI (Kruskal–Wallis, *P* = 0.037, and *P* = 0.008, respectively). SUV_max_ and DOI showed a strong positive correlation (Spearman Rho, correlation coefficient = 0.451, *P* = 0.0003). An increase in SUV_max_ predicted nodal metastasis (Kruskal–Wallis, *P* = 0.017) and poor local control (log rank, *P* = 0.047).

**Conclusion:**

In OSCC, FDG-PET-derived metabolic tumor parameter SUV_max_ serves as a surrogate marker for hypoxia and can be used to predict tumor aggressiveness, with more invasive phenotypes and poorer local control.

## Introduction

Oral squamous cell carcinoma (OSCC) is an aggressive malignancy characterized by local invasiveness and high propensity for early nodal dissemination (1, 2). The importance of depth of invasion (DOI) of the primary tumor and its correlation with the prevalence of lymph node metastasis is now widely recognized. It was implemented into the latest edition of the tumor–node–metastasis (TNM) classification system (3, 4).

Oral squamous cell carcinoma undergoes phenotypic changes to gain migratory and invasive properties through the process of epithelial–mesenchymal transition (EMT) (5, 6). Enrichment in EMT sustains transcription of several matrix metalloproteinases, initiating focal matrix degradation and allowing invasion (7). EMT also enhances the glycolytic phenotype of cancer cells that are exposed to hypoxia (6). Tumor cells increasingly metabolize glucose through glycolysis rather than oxygen-dependent Krebs cycle when exposed to hypoxia. This phenomenon is often referred to as the Warburg effect (8).

Clinically, glucose consumption of tumors can be estimated pretherapeutically by functional nuclear medicine imaging with 18-fluoro-desoxy-glucose positron emission tomography (FDG-PET). Imaging metabolic parameters, such as maximum standard uptake value (SUV_max_), provide *in vivo* quantification of the glucose consumption of a particular tumor (9–11).

As EMT, tumor hypoxia, and the Warburg effect are intricately related (12, 13), we postulated that immunohistochemical expression of hypoxia-related proteins correlates with higher glucose consumption and more invasive phenotype of OSCC. The prognostic relevance of hypoxia in oral cancer has already been reported by other studies before, mostly using immunohistochemical staining for hypoxia biomarker such as hypoxia-inducible factor 1-alpha (HIF1a) and glucose transporter 1 (GLUT1 or other proteins such as CD44) (14–17). However, very few studies have combined immunohistochemical and oncological outcome and functional imaging data in oral cancer (18–21). We therefore examined the role of hypoxia in OSCC comparing clinical, histological, and functional imaging data, therefore creating a histometabolic profile of OSCC. We also assessed its impact on local control and metastatic spread.

## Materials and Methods

### Study Population

After ethical review board approval (protocol number 2016-01799, including amendment dated December 14th, 2018), the charts of consecutive histologically proven OSCC patients were retrospectively assessed. Patients treated from 2007 to 2018 at the Department of Otorhinolaryngology—Head and Neck Surgery, University Hospital Zurich, Switzerland, with available pretherapeutic FDG-PET [combined with computed tomography (CT) or magnetic resonance imaging (MRI)] were included. According to our institutional policy, all patients with primary tumors staged clinically as either ≥ cT3 or ≥ cN2b underwent pretherapeutic FDG-PET (11). Therefore, the study cohort included mainly patients with advanced OSCC stages. Only patients treated with curative intent and without distant metastasis at initial presentation were included. Patients with previous treatment of another head and neck squamous cell carcinoma and/or patients after induction chemotherapy were excluded. Adjuvant radio(chemo)therapy was administered according to the National Comprehensive Cancer Network (NCCN) guidelines after review of final pathology (22).

Detailed data on age, gender, smoking, drinking habits, clinical and pathological stage, DOI, number of lymph nodes dissected, number of positive lymph nodes, local and regional recurrence, distant metastasis, disease-specific survival, and overall survival were obtained. Patients were staged according to the *Union Internationale Contre le Cancer (UICC)*, TNM staging for head and neck cancer, 8th edition 2017 (3).

### Immunohistochemistry and Immunohistochemical Scoring

An automated Ventana BenchMark Ultra (Roche-Ventana Medical System, Tucson, AZ, United States) was used for staining according to the manufacturer’s instructions. Immunohistochemical staining for CD44, for GLUT1, and for HIF1a were performed according to previous studies by our group (4, 23). Positive and negative controls were included in all reactions. Immunohistochemical scoring assessing the whole histological slides was performed by a board-certified pathologist (NJR) and board-certified head and neck surgeon (GBM). For equivocal cases, a consent was reached. If necessary, hematoxylin, and eosin slides were assessed as well. NJR and GBM were blinded to the outcome and further clinicopathological data during the scoring.

CD44 and GLUT1 both showed a distinct membranous staining and were scored in a four-tiered system [negative, weak (1+), moderate (2+), and strong (3+) according to a previous publication (4)]. For GLUT1 (polyclonal antibody, 1:1,000, Millipore), staining of nearby erythrocytes was used as a positive control. HIF1a (antibody clone mgc3, 1:400, Abcam Limited) showed a nuclear staining and was scored with a similar system as GLUT1, ranging from 0 to 2+. A score of 1 + demonstrated at least 10% of tumor cells with nuclear staining.

The hypoxia score was created adding the GLUT1 score (0–3+) and the binarized HIF1a score, therefore ranging from 0 to 4+.

### FDG-PET/CT or FDG-PET/MR Image Acquisition

All patients fasted for at least 4 h prior to the scan. Patients were injected with a standardized dose of 3.5 MBq FDG/kg body weight (analog PET/CT) or a body mass index (BMI)-adjusted FDG dosage protocol (digital PET/CT and PET/MR) (24). All patients had a blood glucose level below 10 mmol/L before imaging. During the uptake time of 1 h, patients rested in a silent, warm, and dimmed environment. Scans were acquired using integrated PET/CT scanners (Discovery VCT, Discovery 690, or Discovery MI, GE Healthcare, Waukesha, WI, United States) or an integrated PET/MR scanner (Signa PET/MR, GE Healthcare). Scans included either a diagnostic CT scan of the neck after administration of iodinated contrast medium or a diagnostic regionalized PET/MR scan of the neck using gadolinium-based contrast medium. Detailed technical acquisition protocols have been published previously (11).

### Metabolic Parameters

The standardized uptake value (SUV) was calculated automatically [activity in volume of interest (VOI)/(injected dose × body weight)]. The SUV_max_ is defined as the hottest voxel within the VOI. SUV_mean_ is defined as the average SUV of voxels within the VOI exceeding 42% of the SUV_max_. The metabolic tumor volume (MTV) is defined as the sum of the volume of voxels with an SUV exceeding a threshold of 42% of the SUV_max_ within the VOI. Total lesion glycolysis (TLG) is defined mathematically as MTV × SUV_mean_. For the analysis of FDG uptake, correct placement of volumes of interest on PET images is ensured by side-by-side reading of the corresponding CT or MR images. A written radiological report by a doubly board-certified nuclear medicine physician/radiologist was available for all FDG-PET/CT or FDG-PET/MR examinations.

### Statistical Analysis

Mean, standard deviation (±SD), standard error of mean (±SEM), median, or interquartile range (IQR) are given for descriptive analysis of continuous variables. For comparison of means, the *t* test was used for normally distributed variables. Alternatively, the non-parametric Kruskal–Wallis test was used. Binary variables were associated in contingency tables using the chi-squared test.

For primary outcome analysis, correlations between continuous variables were assessed using the Spearman rho test. Curve estimations were performed using a linear model not including a constant in the equation. Receiver operating characteristic (ROC) curve was used to select the best cutoff value for SUV_max_ to predict DOI > 10 mm with the 95% confidence interval provided (95% CI). The relative sensitivity and specificity were calculated according to the Bayes’ theorem. For secondary outcome analysis, survival curves were built according to Kaplan–Meier, and the log-rank test was used to compare factors. Finally, a Cox regression model was built to assess survival in multivariable analysis, including all relevant baseline characteristics (Table 1), the hypoxia score, and SUV_max_ of the primary tumor. We did not include DOI in the model because DOI is collinear with T-classification and with SUV_max_ of primary tumor. Inclusion of DOI leads to instability of the model (25). A *P* ≤ 0.05 was considered to indicate statistical significance. Statistical analyses were performed using SPSS 25.0.0.1 software (IBM, Armonk, NY, United States).

**TABLE 1:** Baseline characteristics of study patients.

Variable	All patients No. = 98
**Age (years)**	
Mean (SD)	65.4 (SD 13.3)
**Gender**	
Male	64 (65.3%)
Female	34 (34.7%)
**Smoking**	
Yes	56 (55.4%)
No	45 (44.6%)
**Alcohol**	
Yes	40 (39.6%)
No	61 (60.4%)
**Tumor subsite**	
Oral tongue	41 (41.8%)
Floor of mouth	32 (32.7%)
Upper/lower gum	17 (17.3%)
Other	8 (8.2%)
**T category**	
pT1/pT2	56 (57.1%)
pT3/pT4	42 (42.9%)
**N category**	
pN0	49 (50.0%)
pN+	49 (50.0%)

*No., number and SD, standard deviation.*

## Results

### Patient and Tumor Characteristics

A total of 98 consecutive patients were included in the study. The mean age at diagnosis was 65.4 (SD, 13.3) years. There was a clear male predominance with 64 (65.3%) male and 34 (34.7%) female patients. Forty-one (41.8%) had squamous cell carcinomas of the oral tongue, 32 (32.7%) of the floor of mouth, and 17 (17.3%) of the gum. Eight (8.2%) tumors were located elsewhere (retromolar trigone, buccal mucosa). Slightly more than half (56, 57.1%) of the patients had pT1–pT2 tumors, while 42 (42.9%) had pT3–pT4 tumors. All patients underwent pathological assessment of nodal status, 12 (12.2%) of these with sentinel lymph node biopsy, the remaining 86 (87.8%) with neck dissection. Of those, 12 (12.2%) had bilateral neck dissection. Mean number of dissected nodes for sentinel, unilateral, and bilateral neck dissection was 2.8 (±SEM 0.6), 25.8 (±SEM 1.2), and 48.4 (±SEM 4.1), respectively. Nodal status was positive (pN+) in 49 patients (50%), of which 16 (16.5%) were staged with pN1, 20 (20.4%) pN2a–pN2b, and 13 (13.2%) pN2c–pN3 categories (Table 1).

In total, 59 (60.2%) patients received adjuvant radiotherapy with a mean local dose of 60.5 Gy (±SEM 1.2) and 52.3 Gy (±SEM 2.7) for the nodal basin. 20 (20.4%) patients received concomitant chemotherapy. Mean follow-up time of the cohort was 24.2 months (±SEM 2.1).

### A High Primary Tumor SUV_max_ Correlates With Deep Invading Tumors

**FIGURE 1:**
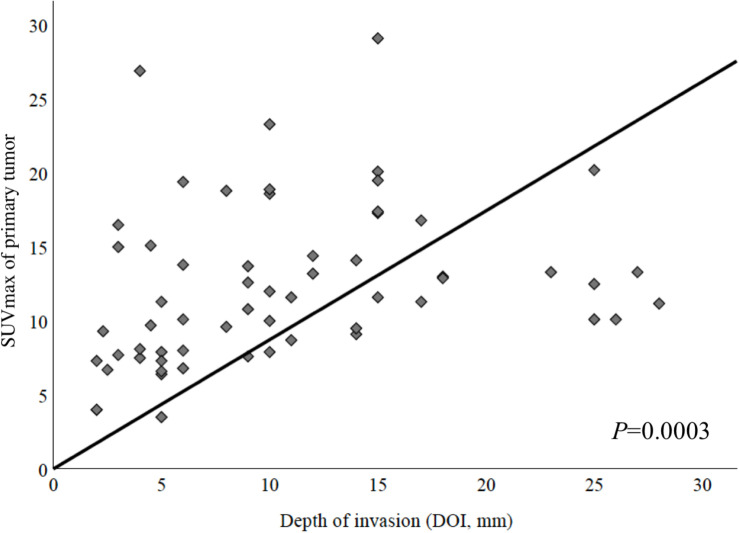
Correlation between maximum standardized uptake value (SUV_max_) of primary tumor and depth of invasion (DOI). These parameters showed a strong positive correlation (Spearman Rho, correlation coefficient = 0.451, *P* = 0.0003).

In a first step, we examined whether metabolic tumor imaging showed a correlation with histological DOI of the primary tumor. DOI and SUV_max_ showed a strong positive correlation (Figure 1, Spearman Rho, correlation coefficient = 0.451, and *P* = 0.0003). A similar observation could be made when correlating DOI and MTV (Spearman Rho, correlation coefficient = 0.486, *P* = 0.004, not shown). TLG did not correlate significantly with DOI (*P* > 0.05, not shown).

Various cutoff values for SUV_max_ were tested. Using ROC curves, the best potential cutoff value for the pretherapeutic SUV_max_ of primary tumor was determined to be 9.5 for the highest sensitivity and 14.5 for the highest specificity, respectively, [area under the curve (AUC), 70.8% (95% CI, 57.8–83.8%); *P* = 0.007; Table 2].

**TABLE 2:** Diagnostic accuracy of SUV_max_ in prediction of depth of invasion.

Variable	Depth of invasion < 10 mm		
SUVmax primary tumor < 9.5	TP	FP	Sensitivity
	21	19	87.5%
	FN	TN	Specificity
	3	17	47.2%
SUVmax primary tumor > 14.5	TP	FP	Sensitivity
	7	9	29.2%
	FN	TN	Specificity
	17	27	75.0%

*SUV_*max*_, standardized maximum uptake value; TP, true positive; FP, false positive; FN, false negative; and TN, true negative.*

### A High Primary Tumor SUV_max_ Is Associated With Positive Neck Disease

Second, we compared the distributions of SUV_max_ of primary tumors among patients with positive and negative nodal disease (pN + vs. pN0).

The median SUV_max_ of primary tumor was 13.2 (IQR, 10.1–17.1) and 11.0 (IQR, 7.8–15.1) in the nodal positive and negative group, respectively.

**FIGURE 2:**
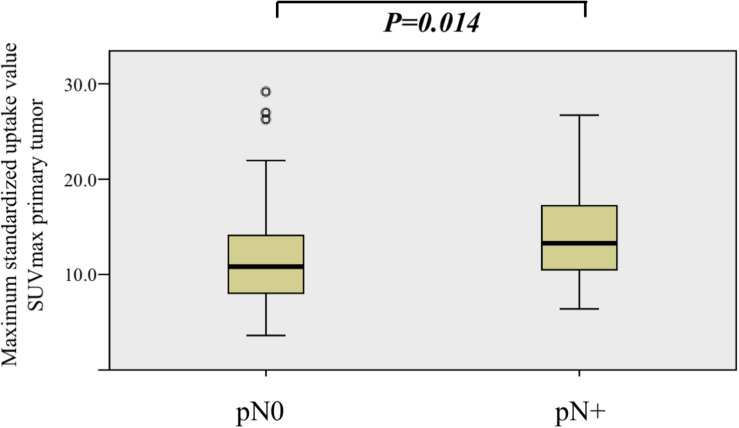
Comparison of maximum standardized uptake value (SUV_max_) value distribution among nodal positive (pN+) and nodal negative (pN0) patients. SUV_max_ was significantly higher in the nodal positive (pN+) group (Kruskal–Wallis test, *P* = 0.013).

When comparing the distribution of SUV_max_ among pN+ and pN0, there was a statistically significant difference between the two groups (Kruskal–Wallis test, *P* = 0.013, Figure 2).

### Correlation Between SUV_max_, Immunohistochemical Surrogates for Hypoxia, and Depth of Invasion

Furthermore, we analyzed immunohistochemical stainings for the glucose transporter protein GLUT1 in primary tumor samples. Patients showing a strong expression of GLUT1 (score > 2+) had a significantly higher SUV_max_ (Kruskal–Wallis test, *P* = 0.028) than patients with low/moderate (score ≤ 2+) expression of GLUT1. For MTV and TLG, there was no statistical difference (Kruskal–Wallis test, *P* = 0.107 and *P* = 0.160), respectively. Strong expression of GLUT1 was also associated with greater DOI (Kruskal–Wallis test, *P* = 0.008).

Positive expression of HIF1a (score ≥ 1+) was associated with significantly greater DOI (Kruskal–Wallis test, *P* = 0.018), which means deep invading tumors. The expression of HIF1a alone did not correlate with SUV_max_, MTV, or TLG (Kruskal–Wallis test, *P* = 0.184, *P* = 0.771, and *P* = 0.134, respectively).

CD44 expression was not associated with any metabolic parameter and/or DOI (Kruskal–Wallis test, *P* > 0.05, not shown).

### Hypoxia Score for Enhanced Accuracy

**FIGURE 3:**
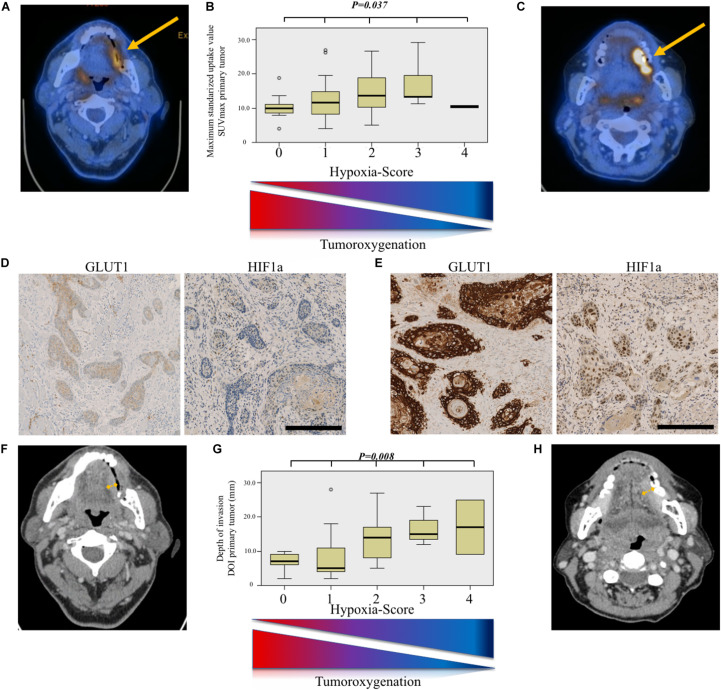
Hypoxia-score model for prediction of aggressive tumor phenotype. **(A)** Axial 18-fluoro-desoxy-glucose positron emission tomography/computed tomography (FDG-PET/CT) image showing a weakly metabolically active tumor (SUV_max_ = 5.1). **(B)** Comparison of maximum standardized uptake value (SUV_max_) value distribution according to hypoxia score. The higher the score, the higher the SUV_max_ (Kruskal–Wallis test, *P* = 0.037). **(C)** Axial FDG-PET/CT image showing a strongly metabolically active tumor (SUV_max_ = 19.4). **(D)** Immunohistochemical staining for glucose transporter 1 (GLUT1) and hypoxia-inducible factor 1-alpha (HIF1a) showing a weak membranous and nuclear staining, respectively, resulting in a low hypoxia score. Scale bar, 250 μm. **(E)** Immunohistochemical staining for GLUT1 and HIF1a showing a strong membranous and nuclear staining, respectively, resulting in a high hypoxia score. Scale bar, 250 μm. **(F)** Axial contrast-enhanced computed tomography (CT) image in the same patient as **(A)** showing a tumor with modest depth of invasion (DOI). Definitive DOI on histopathology was 2 mm. **(G)** Comparison of depth of invasion (DOI, mm) value distribution according to hypoxia score. The higher the score, the deeper the tumoral infiltration (Kruskal–Wallis test, *P* = 0.008). **(H)** Axial contrast-enhanced computed tomography (CT) image in the same patient as **(C)** showing a tumor with deeper invading tumor. Definitive DOI on histopathology was 6 mm.

To optimize the likelihood of tissue-based tumor hypoxia detection, we created a hypoxia score by combining the scores of the immunohistochemical staining for GLUT1 and the binarized HIF1a, both known to be upregulated under hypoxic conditions. As shown in Figure 3, for both SUV_max_ and DOI, the association showed a dose–response relationship: the higher the hypoxia score, the higher the SUV_max_ and the deeper the tumor infiltration (Kruskal–Wallis test, *P* = 0.037, and *P* = 0.008, respectively).

### Impact of SUV_max_, DOI, and Hypoxia-Related Protein Expression on Oncological Outcomes

There were 14 (14.3%) local recurrences in the cohort, whereas 16 (16.3%) patients had regional recurrence, and 14 (14.3%) had distant metastasis. Fifteen (15.3%) patients died from their tumor.

**FIGURE 4:**
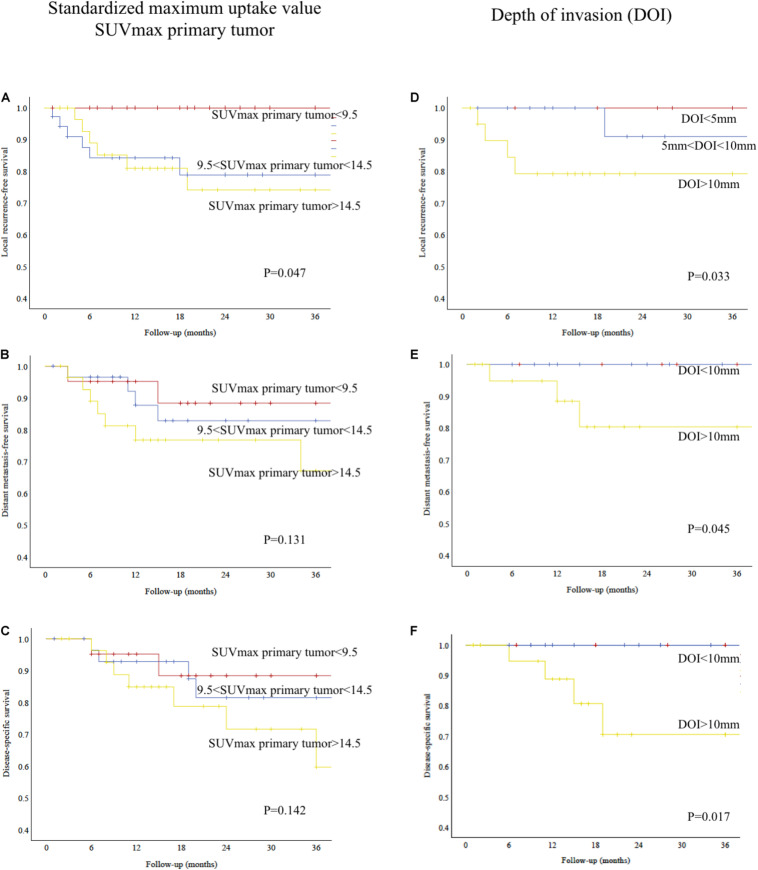
**(A)** Kaplan–Meier analysis showing relative survival according to SUV_max_ of primary tumor. High SUV_max_ predicted poorer local recurrence-free survival (log-rank test, *P* = 0.047). **(B)** Kaplan–Meier analysis showing distant metastasis-free survival according to SUV_max_ of primary tumor (log-rank test, *P* = 0.131). **(C)** Kaplan–Meier analysis showing disease-specific survival according to SUV_max_ of primary tumor (log-rank test, *P* = 0.142). **(D)** Kaplan–Meier analysis showing relative survival according to depth of invasion (DOI). Patients with deep tumors had a poorer local recurrence-free survival (log-rank test, *P* = 0.033). **(E)** Kaplan–Meier analysis showing distant metastasis-free survival according to depth of invasion (DOI). Patients with deep tumors had a poorer distant metastasis-free survival (log-rank test, *P* = 0.045). **(F)** Kaplan–Meier analysis showing disease-specific survival according to depth of invasion (DOI). Patients with deep tumors had a poorer disease-specific survival (log-rank test, *P* = 0.017).

As shown in Figure 4A, local recurrence-free survival was significantly worse for patients with higher SUV_max_ of the primary tumor (log-rank test, *P* = 0.047). For regional recurrence-free, distant metastasis-free, and disease-specific survival, there was no statistically significant difference (log-rank test, *P* = 0.140, not shown; *P* = 0.131, Figure 4B; and *P* = 0.142, Figure 4C; respectively).

As shown in Figures 4D–F, local recurrence-free survival, distant metastasis-free, and disease-specific survival were significantly worse for patients with deep invading tumors (log-rank test, *P* = 0.033, Figure 4D; *P* = 0.045, Figure 4E; and *P* = 0.017, Figure 4F; respectively). There was no statistically significant difference for regional recurrence-free survival (log-rank test, *P* = 0.275, not shown).

When directly correlating expression of GLUT1, HIF1a, and hypoxia score with oncological outcomes, patients showing a strong expression of GLUT1 (score > 2+) had a poorer local recurrence-free survival (log-rank test, *P* = 0.050, not shown). Similarly, patients with a high tissue-based hypoxia score (>3) had a poorer local recurrence-free survival (log-rank test, *P* = 0.031, not shown).

For regional recurrence-free, distant metastasis-free, and disease-specific survivals, expression of GLUT1, HIF1a, and hypoxia score did not predict survival (log-rank test, *P* > 0.05, not shown).

### Multivariable Cox Regression Analysis of Oncological Outcomes

We finally performed a multivariable Cox regression analysis including all relevant factors listed in Table 1, the hypoxia score, and SUV_max_ of the primary tumor.

For local recurrence-free survival, positive nodal disease (pN + vs. pN0) and SUV_max_ of primary tumor were independent predictors of survival (*P* = 0.036 and *P* = 0.046, respectively). All other factors were not independent predictors (*P* > 0.05).

For regional recurrence-free survival, positive nodal disease (pN + vs. pN0) was the sole independent predictor of recurrence (*P* = 0.014). All other factors were not independent predictors (*P* > 0.05).

For distant metastasis-free survival, advanced T classification (pT3–T4 vs. pT2–T1) and positive nodal disease (pN + vs. pN0) were independent predictors (*P* = 0.013 and *P* = 0.031, respectively). All other factors were not independent predictors (*P* > 0.05).

For disease-specific survival, positive nodal disease (pN + vs. pN0) was also the sole independent predictor (*P* = 0.034). All other factors were not independent predictors (*P* > 0.05).

## Discussion

This study evaluates in a cohort of OSCC the correlation between preoperative FDG-PET and outcome through histometabolic tumor imaging of hypoxia. Our study provides some important insights in OSCC with analysis of clinical, histological, and functional nuclear medicine imaging data, therefore creating a histometabolic profile of OSCC.

A hypoxia-related protein overexpression correlated with a higher *in vivo* uptake of glucose, estimated by SUV_max_ on preoperative FDG-PET. This allowed us for definition of a hypoxia score, correlating with DOI of the tumor. This information impacted on local control and metastatic spread in OSCC. As shown in our multivariable analysis, nodal involvement is the most important prognostic factor in OSCC, including for local recurrence-free survival, in concordance with previous publications (26, 27). Therefore, prehistological estimation of tumor aggressiveness and nodal status is very important.

When FDG-PET was first added to the staging process in head and neck squamous cell carcinoma, it improved the nodal classification (28), provided a more accurate detection of distant metastasis (29) and synchronous primary tumors (30), and increased the detection rate of occult primaries (31). Furthermore, it is used to assess local (32) and regional (33) tumor response after chemoradiation. These studies mostly used FDG-PET to evaluate the presence or absence of metabolic activity in a binary manner.

In recent years, studies have examined the potential of quantification of functional nuclear medicine imaging data, using parameters such as maximum SUV_max_, MTV, TLG, or further mathematically derived value such as textural index. Their role in predicting radio-oncological outcomes has now also been demonstrated (9, 34–38). However, a growing body of evidence shows that these data could also be used as a predictive tool for outcomes deemed to be surgical (39), such as occult lymph node metastasis (11), bone infiltration in OSCC (40), or organ preservation in laryngeal and hypopharyngeal cancer (41, 42). Our study reinforces and confirms the prognostic role of SUV_max_ as a surrogate marker for surgical outcomes. We showed that SUV_max_ was an independent predictor of local recurrence-free survival in multivariable analysis. Furthermore, SUV_max_ strongly correlated with DOI, which is a major staging variable, recently integrated into the last edition of the UICC Staging System (3).

A DOI > 10 mm represents a crucial cutoff point from the anatomical and biological points of view in oral tongue and floor of mouth tumors since it marks the passage from the intrinsic to the extrinsic musculature (43). As such, it was implemented by the 8th edition of TNM Staging of UICC as the marking point between early (pT1–2) and advanced disease (pT3–4) (3). The importance of DOI > 10 mm has been also underlined in the survival analyses in this study (Figures 4D–F).

According to our ROC analysis, a SUV_max_ of primary tumor < 9.5 was the optimal cutoff to largely rule out DOI > 10 mm. On the other hand, a SUV_max_ > 14.5 predicted DOI > 10 mm with a rather good specificity. These data could be of adjunctive value in the everyday clinical practice, considering that tumors with DOI > 10 mm require compartment tongue surgical approach (44, 45).

First described by Otto Warburg (8), the ability of tumor cells to survive in an hypoxic environment is gained by promoting a shift toward glycolytic reductive metabolism rather than oxygen-dependent Krebs cycle (oxidative metabolism). Through increased production of reactive oxygen species (ROS) stimulated by hypoxia, upregulation of one of the main activators of the Warburg effect, HIF1a, is promoted (46). This allows adaptation to hypoxia by increasing GLUT1 expression, glycolysis, and lactate production (47). This deregulation in cellular energetics is associated with an induction of EMT through downregulation of E-cadherin and upregulation of twist1 and vimentin (6, 48). Furthermore, an invasive phenotype is promoted through matrix metalloproteinase upregulation (7). Some data indicate that increased SUV_max_ is associated with increased tissue necrosis assessed by, e.g., cleaved caspase 3 staining (49), further supporting the idea of an association between hypoxia and increased SUV_max_ by FDG-PET.

Therefore, our results seem logical and corroborate the findings from previous studies in a larger cohort (18–21). In studies with 36 OSCC and 33 T2-oral tongue cancer patients, a correlation of the primary tumor’s SUV_max_ with GLUT1 and HIF1a expression was found (18, 19). In the first study by Yamada et al. (18), a high SUV_max_ was associated with higher T classification but not nodal status. Correlation with DOI was not performed, which, at the time of their study, was not yet part of the TNM staging system (3, 18). The second study by Han et al. reported that SUV_max_ correlated with hypoxic status assessed by HIF1a and GLUT1 and was predictive of survival in OSCC (19). Interestingly, despite similar results, the data from all of the studies were based on expression analysis of different tissue specimen (biopsy, tissue microarray, and whole slide stainings of the resection specimen in the current study).

Some smaller studies in OSCC did not show any correlation between SUV_max_, GLUT1, and HIF1a. Yokobori et al. showed in 27 early stage OSCCs a correlation between SUV_max_, T stage, and microvessel density but not with GLUT1 (20). In a further study with 31 OSCC patients, SUV_max_ and GLUT1 did not show any correlation, although both of them were predictors of outcome (21).

In other head and neck tumor sites, there are some divergent data as well. In a cohort of oropharyngeal and hypopharyngeal cancer patients, a correlation between SUV_max_, GLUT1, and HIF1a expression was found (38). However, in a further study done mostly in oropharyngeal cancer patients, high GLUT1 expression correlated with EGFR expression and p16 negative status but not with SUV_max_ of the primary tumor (50). In recurrent head and neck squamous cell carcinoma, GLUT1 expression was elevated but did not correlate with SUV_max_ of the primary tumor (51).

Our study has some drawbacks due its retrospective nature not allowing a definitive statement about clinical applicability and the lack of mechanistic demonstration. Furthermore, the low number of events may have led to a beta error in some analyses. Finally, future studies shall examine the correlation between FDG-PET-derived hypoxia estimation and classical hypoxia tracers such as 18F-fluoromisonidazole [(^18^F)-FMISO]-PET, which was shown to predict therapeutic response in clinical studies (52–54). Initial prospective and dynamic data could thereby show an association of HIF1a expression and an increased (^18^F)-FMISO tumor uptake, indicating hypoxic conditions in a cohort of head and neck squamous cell carcinoma (HNSCC). However, only two carcinomas from the oral cavity were included in this study (55). Another study with 15 HNSCC patients did not show any correlation between immunohistochemical markers (including GLUT1 and HIF1a), FDG-PET parameters, and (^18^F)fluoroerythronitroimidazole [(^18^F)FETNIM]-PET, another hypoxia/perfusion tracer (56).

In conclusion, a quantification of the FDG uptake under standardized conditions can be used as a surrogate for tumor aggressiveness, since it correlates with the Warburg effect, tumor hypoxia, EMT, and invasiveness. It allows for definition of a histometabolic profile, allowing prehistological prediction of DOI and nodal status, the latter being the most important prognostic factor in OSCC. This information may be useful in surgical planning, indication for adjuvant radiotherapy, approach to the neck, and patient counseling.

## Data Availability Statement

The datasets generated for this study can be obtained upon reasonable request by email to the corresponding author.

## Ethics Statement

The studies involving human participants were reviewed and approved by Kantonale Ethikkommission Zürich. The patients/participants provided their written informed consent to participate in this study.

## Author Contributions

GM and NR formulated the basic study idea. GM conducted patients search. MH extracted the data related to nuclear imaging. NR and GM performed immunohistological staining and scoring. GM extracted the patient-related data, performed statistical analysis, built the figures, and wrote the first draft of the manuscript. MB, PS, MH, and NR edited and reviewed the manuscript. GM, MB, PS, MH, and NR have participated substantially to the study and approved the final version of the manuscript. All authors contributed to the article and approved the submitted version.

## Conflict of Interest

MH is a recipient of grants from GE Healthcare and received an Alfred and Annemarie von Sick grant for translational and clinical cardiac and oncological research. The remaining authors declare that the research was conducted in the absence of any commercial or financial relationships that could be construed as a potential conflict of interest.
